# Long-term follow-up of a bilateral acute posterior multifocal placoid pigment epitheliopathy following COVID-19 infection: a case report

**DOI:** 10.1186/s12348-023-00382-x

**Published:** 2024-01-04

**Authors:** Casper Lund-Andersen, Oliver Niels Klefter, Miklos Schneider

**Affiliations:** 1 Department of Ophthalmology, Rigshospitalet Glostrup, Valdemar Hansens Vej 1-23, 2600 Glostrup, Denmark; 2 Department of Clinical Medicine, University of Copenhagen, Copenhagen, Denmark; 3 Department of Ophthalmology, Semmelweis University, Budapest, Hungary

**Keywords:** Acute posterior multifocal placoid pigment epitheliopathy, APMPPE, Relentless placoid chorioretinitis, RPC, COVID-19, SARS-CoV-2, Coronavirus, Prednisolone, Steroid, Adalimumab

## Abstract

**Background:**

Acute posterior multifocal placoid pigment epitheliopathy (APMPPE) is a rare inflammatory eye disorder that is characterized by the presence of multiple placoid lesions in the posterior pole of the eye. Relentless placoid chorioretinitis (RPC) is an inflammatory chorioretinopathy that combines clinical features of APMPPE and serpiginous chorioretinitis, which is a progressive condition with a high risk of visual disability. Patients with COVID-19 can develop various ocular manifestations, however, there have been limited reports of APMPPE and RPC associated with the infection.

We report a case of a patient who developed APMPPE after a COVID-19 infection and subsequently progressed into RPC.

**Case presentation:**

A 17-year-old male presented with a one-week history of painless gradual visual loss in both eyes. Two months prior to the visual symptoms, the patient had a SARS CoV-2 infection, confirmed by polymerase chain reaction test. Clinical findings with fundoscopy, optical coherence tomography and fluorescein angiography were consistent with APMPPE. Due to the severely affected vision in both eyes, the patient was started on 50 mg oral prednisolone daily, after which vision began to improve rapidly. Two months after symptom onset during steroid taper, the impression of continued inflammatory activity and new lesions in the retinal periphery of both eyes suggested RPC. Adalimumab 40 mg every other week was initiated with 12.5 mg prednisolone daily followed by slow tapering. Vision improved and five months after the start of the adalimumab treatment, the steroid was discontinued and there were no signs of active inflammation. The patient has been followed for a total of 21 months since presentation, had full visual recovery and good tolerance of the immunosuppressive treatment.

**Conclusion:**

COVID-19 might cause long-lasting activity of APMPPE. The scarcity of reports compared with the number of confirmed COVID-19 infections worldwide suggests a rare entity. The association of APMPPE with a variety of infections may suggest a common immunological aberrant response that might be triggered by various factors. Further examinations and case reports are needed to understand the role of biological therapy in the treatment of such cases.

**Supplementary Information:**

The online version contains supplementary material available at 10.1186/s12348-023-00382-x.

## Introduction

Acute posterior multifocal placoid pigment epitheliopathy (APMPPE) is a rare inflammatory eye disorder that is characterized by the presence of multiple placoid lesions in the posterior pole of the eye. Although the exact pathogenesis of APMPPE is not fully understood, it is thought to be an immune-mediated disorder that may be triggered by viral infections [1].

Relentless placoid chorioretinitis (RPC) is an inflammatory chorioretinopathy that combines clinical features of APMPPE and serpiginous chorioretinitis (SC) [2]. The latter is a progressive condition with a high risk of visual disability [3].

Since the emergence of the COVID-19 pandemic, there have been reports of patients with COVID-19 developing various ocular manifestations, including conjunctivitis, anterior uveitis, and retinal changes [4, 5] However, there have been limited reports of APMPPE and RPC associated with COVID-19.

Here, we report a case of a patient who developed APMPPE after a COVID-19 infection and subsequently progressed into RPC.

## Case presentation

A 17-year-old Caucasian male presented at our outpatient clinic with a one-week history of painless gradual visual loss in both eyes. Two months prior to the visual symptoms, the patient had a SARS CoV-2 infection (B.1.1.7 subtype), confirmed by polymerase chain reaction (PCR) test. He had mild symptoms and was otherwise healthy and reported no other concurrent infections or any other extraocular/systemic symptoms.

**Fig. 1:**
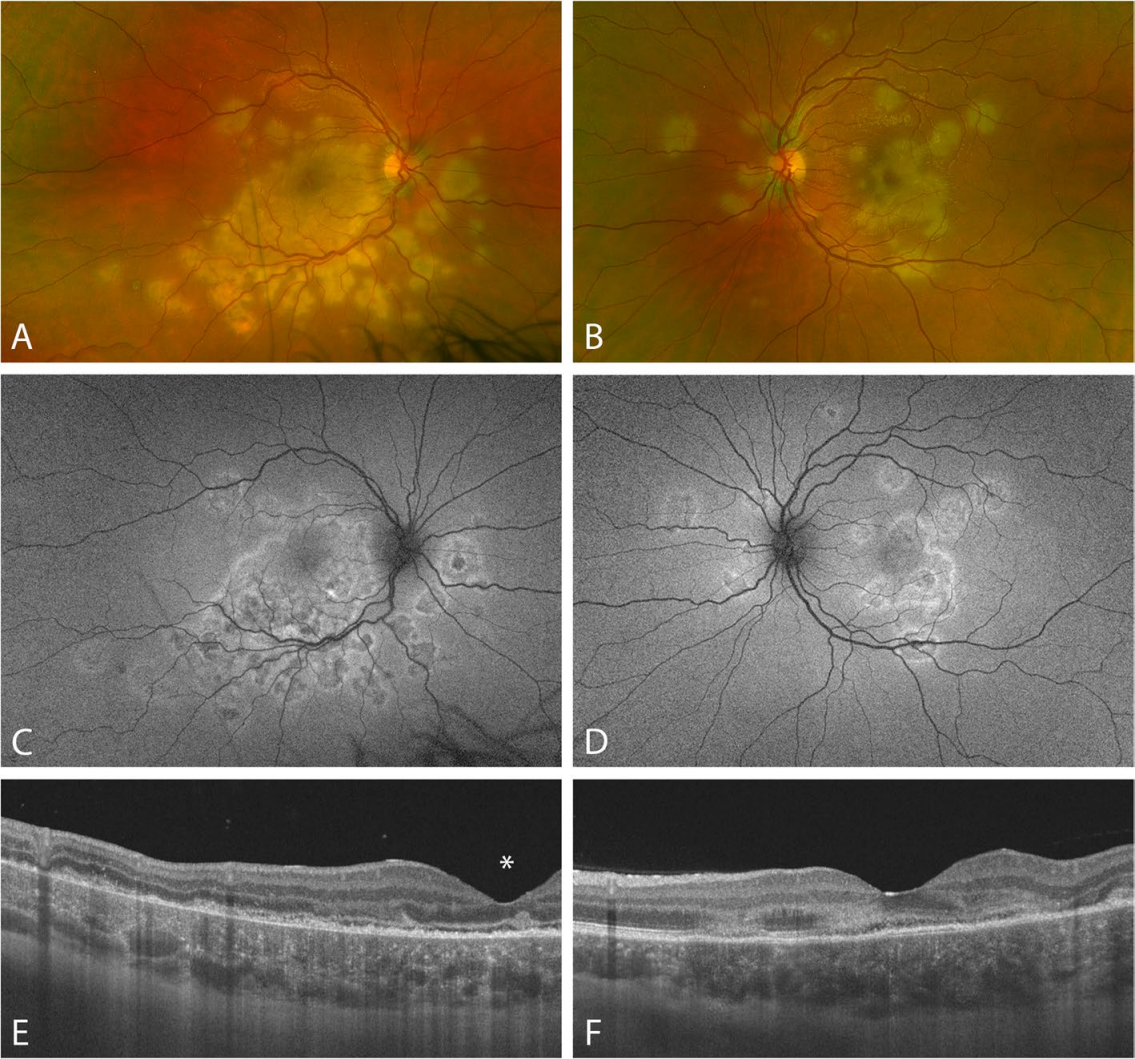
Multimodal imaging at first presentation. **A**, **B**: Wide-field color fundus scanning laser ophthalmoscopy (Optos California, Optos Inc., Marlborough, MA, USA). **C**, **D**: Wide-field fundus autofluorescence imaging (Optos California). **E**, **F**: Optical coherence tomography imaging (Topcon DRI OCT Triton, Topcon Corp., Tokyo, Japan). Note the position of the fovea (asterisk, *), which could not be positioned centrally due to poor fixation

**Fig. 2:**
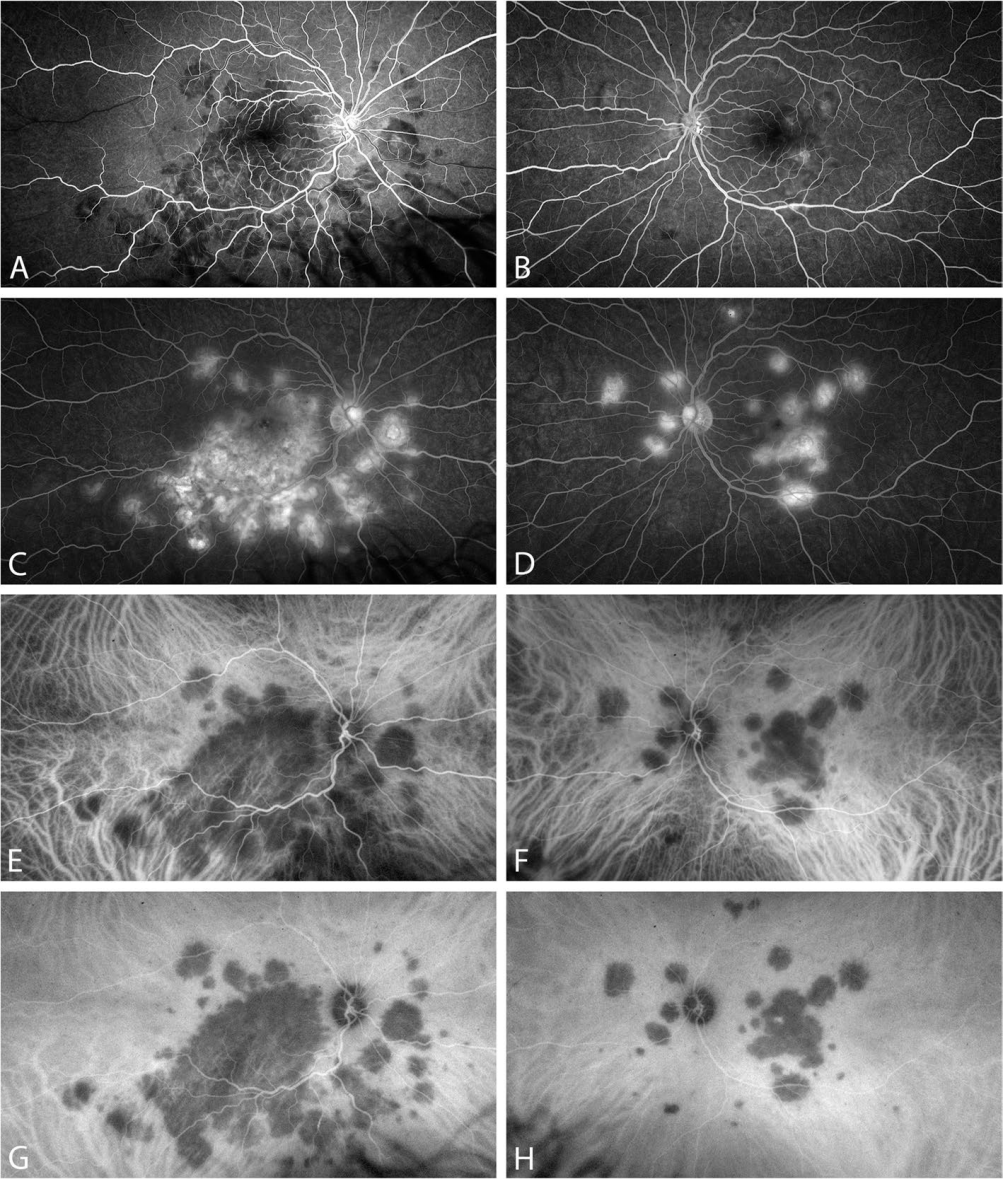
Angiography at first presentation. **A**-**D**: Fluorescein angiography; **A**, **B**: early phase; **C**, **D**: late phase. **E**–**H**: Indocyanine-green angiography; **E**, **F**: early phase; **G**, **H**: late phase. (Optos California, Optos Inc., Marlborough, MA, USA)

On first presentation, best corrected visual acuity (BCVA) was counting fingers on the right eye and 0.1 Snellen on the left eye. Slit lamp biomicroscopy revealed 0.5+ cells in the vitreous in both eyes. Dilated fundus examination revealed creamy confluent yellow-white lesions in the macula in both eyes (Fig. 1. A, B). Fundus autofluorescence imaging (FAF) revealed slightly hypoautofluorescent spots and patches with faint hyperautofluorescent halos around them (Fig. 1. C, D). Optical coherence tomography (OCT) showed areas with hyperreflectivity of the outer retinal layers with absence of the ellipsoid zone and the impression of a flat serous retinal detachment. The scans in the choroid under the lesions showed hyperreflectivity and the disorganization of the regular vessel structure, suggesting chorio-retinal infiltrates (Fig. 1. E, F). Fluorescein angiography (FA) showed early hypofluorescence in the affected areas (Fig. 2. A, B) with hyperfluorescence in late phases (Fig. 2. C, D), indocyanine-green angiography (ICGA) showed both early and late hypocyanescence (Fig. 2. E–H). There were no neurological symptoms or signs. The clinical findings were consistent with acute posterior placoid pigment epitheliopathy (APMPPE). A full diagnostic workup (hematology, serum biochemistry, renal and liver function tests, serum angiotensin convertase enzyme, hemoglobin A1c) was initiated including blood tests for additional potential relevant infectious causes (tuberculosis, treponema), all of which came back negative. Chest x-ray showed no pathologies.

**Fig. 3:**
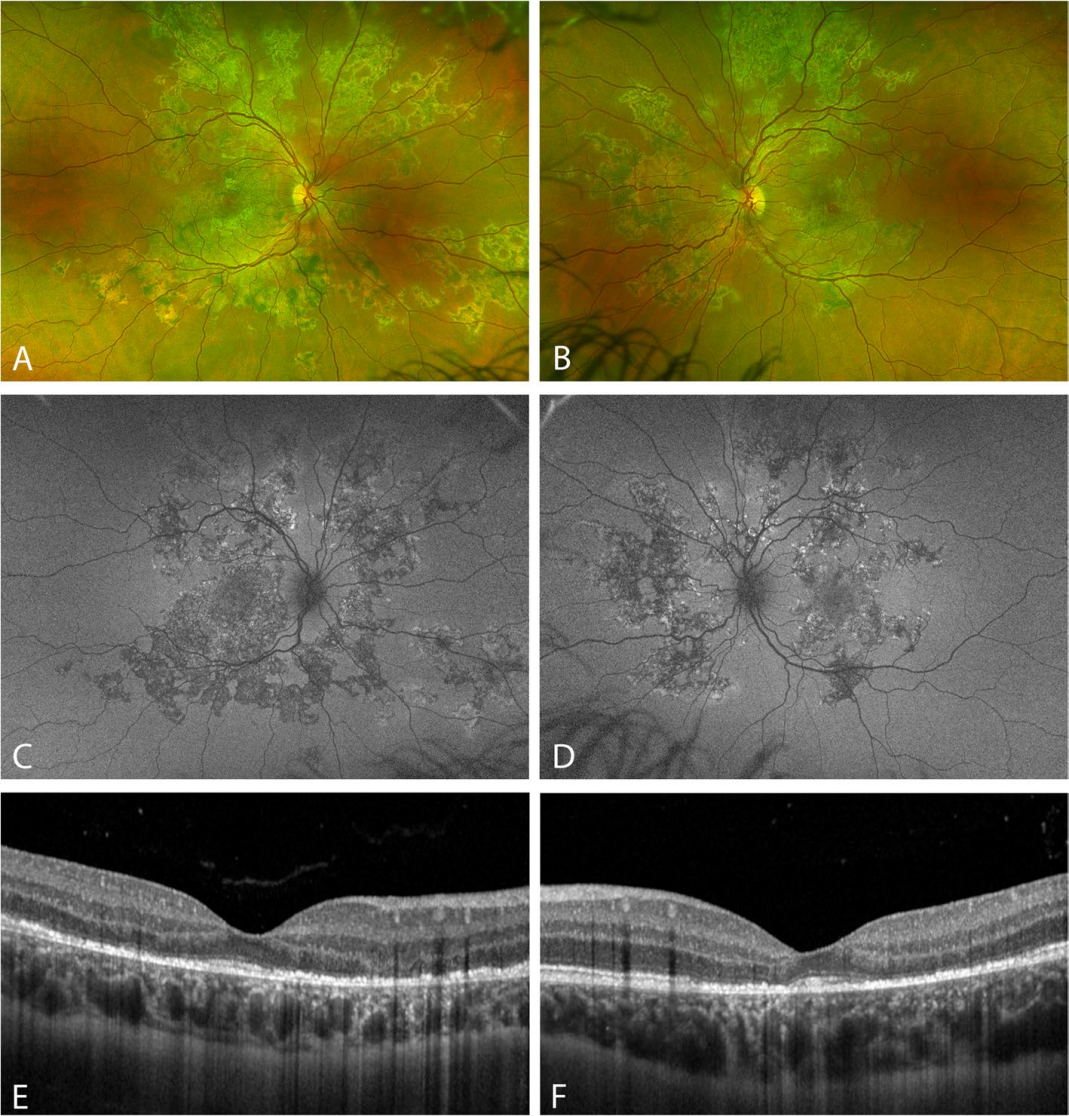
Imaging at 2 months after symptom onset. **A**, **B**: Wide-field color fundus scanning laser ophthalmoscopy (Optos Silverstone, Optos Inc., Marlborough, MA, USA). **C**, **D**: Wide-field fundus autofluorescence imaging (Optos Silverstone). **E**, **F**: Optical coherence tomography imaging with enhanced depth imaging mode (Heidelberg Spectralis OCT, Heidelberg Engineering Gmbh, Heidelberg, Germany)

**Fig. 4:**
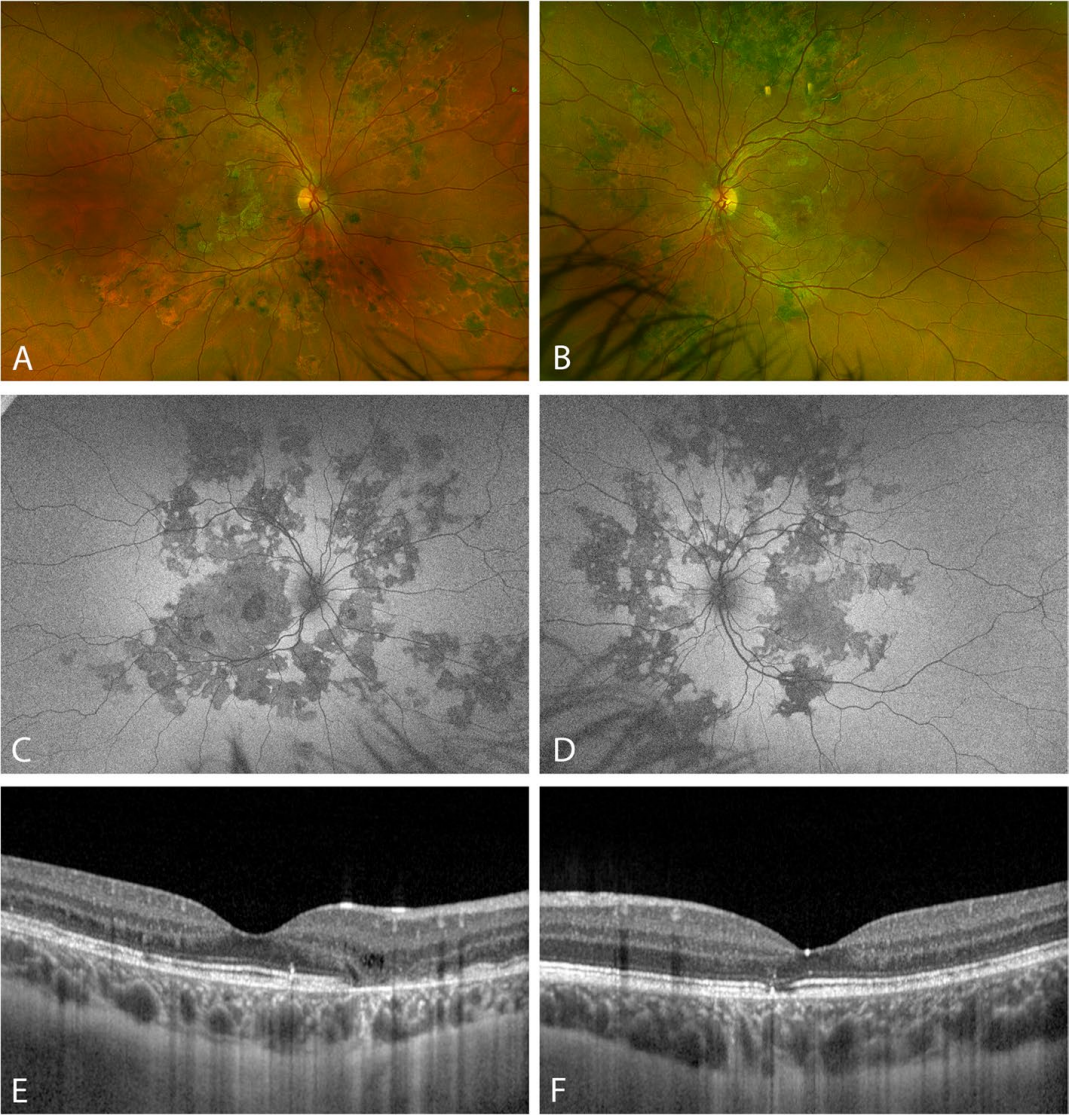
Imaging at 21 months after symptom onset. **A**, **B**: Wide-field color fundus scanning laser ophthalmoscopy (Optos Silverstone, Optos Inc., Marlborough, MA, USA). **C**, **D**: Wide-field fundus autofluorescence imaging (Optos Silverstone). **E**, **F**: Optical coherence tomography imaging with enhanced depth imaging mode (Heidelberg Spectralis OCT, Heidelberg Engineering Gmbh, Heidelberg, Germany)

Due to the severely affected vision in both eyes, the patient was started on 50 mg oral prednisolone daily, after which vision began to improve rapidly. At the next follow-up, one week after the initial presentation, BCVA was 0.5 on the right and 0.15 on the left eye. One week later, we began gradual tapering of the prednisolone dose. Over the course of the next few weeks, vision improved to 0.6 on both eyes and the lesions began to appear more demarcated and pigmented with paracentral atrophy. Two months after symptom onset, when prednisolone had been tapered to 12.5 mg per day, the impression of continued inflammatory activity and new lesions in the retinal periphery of both eyes suggested relentless placoid chorioretinitis (RPC) (Fig. 3.). This prompted a rheumatologic consultation with the aim of switching the patient to steroid-sparing therapy. Simultaneously, an MRI scan was performed, that showed no pathologies and hepatitis B and C tests were negative. Based on the rheumatological consultation and a discussion with the patient, adalimumab treatment with 40 mg every other week was initiated with a continued dose of 12.5 mg prednisolone daily followed by slow tapering of the steroid. Over the course of the next few months, vision improved to 0.9 on both eyes. At month 7 after baseline, the patient had a second PCR-confirmed SARS CoV-2 infection (subtype unknown), that had no further impact on the ocular course. Five months after the start of the adalimumab treatment (at month 9 after onset), the steroid was discontinued and there were no signs of active inflammation. The patient has been followed for a total of 21 months since presentation, at the last visit, 12 months after the discontinuation of the systemic steroid, there was no visible activity suggesting inflammation (Fig. 4.) and vision was 0.9 on the right and 1.0 on the left eye with no subjective visual complaints and with good tolerance of the immunosuppressive treatment. Once the patient has demonstrated no relapses on adalimumab monotherapy for 18–24 months, we plan to increase the dosing interval, thus tapering the immunosuppressive treatment under close clinical observation.

## Discussion

Since the outbreak of COVID-19, there have been several reports of ocular manifestations associated with the disease. Reported ocular symptoms include conjunctivitis [6], anterior uveitis, dynamic retinal vascular abnormalities such as Paracentral Acute Middle Maculopathy (PAMM) or Acute Macular Neuroretinopathy (AMN) and rarely, inflammation of the retina or choroid [4, 7].

We report a case of a severe bilateral macular dysfunction that manifested 2 months after PCR confirmation of a COVID-19 infection. The time passed between the infection and the manifestation of the eye symptoms may seem rather long, but since there was no other event in the patient’s medical history, and chest x-ray, MRI scans and laboratory tests for other potentially relevant infectious origins (such as tuberculosis, treponema, hepatitis) came back negative, we have no reason to suspect any other concurrent causes. Additionally, it is not impossible that the patient’s disease has started earlier with subclinical symptoms that progressed over weeks, and he only noticed them when his central vision became affected. APMPPE is bilateral (simultaneously or sequentially within a few days) in most patients, and the timing of onset for the disease is variable. Typically, it occurs within weeks to months following an antecedent infection with some of the patients having recurrences, most of which present within the first 6 months [8].

To the best of our knowledge, this is the third description of APMPPE following COVID-19 infection, the second bilateral case with the full description of the clinical course and treatment, and the first with long-lasting activity where progression to RPC was observed and where long-term steroid treatment and biological therapy was necessary. Olguín-Manríquez et al. documented a unilateral case of APMPPE occurring six weeks after a COVID-19 infection. However, the article does not mention treatment, or disclose the final outcome [9]. Fisher et al. reported a bilateral case that started 2 weeks after fever, cough, shortness of breath, myalgias and subsequent positive PCR test for SARS-CoV2. Their case had a 5-week course and complete resolution of the lesions and subretinal fluid following prednisone therapy with a starting dose of 60 mg followed by gradual tapering, and 20/20 final visual acuity on both eyes [10].

Ampiginous chorioretinitis shares similar demographics, angiographic and clinical features, and clinical course with RPC and the two entities most likely represent variants of the same disease [11, 12]. According to our knowledge, there are two cases of ampiginous chorioretinitis described in connection with COVID-19 infection. Carvalho et al. reported a bilateral case one week following a confirmed SARS-CoV-2 infection. They treated the patient with prednisolone 0.5 mg/kg daily with slow tapering over 2 months and the patient reached 20/20 vision in both eyes [13]. Tom et al. described a bilateral ampiginous chorioretinitis one week following a presumed SARS-CoV-2 infection, diagnosed with IgG antibody test without PCR. Their patient received a 60 mg oral prednisone starting dose, the retinal lesions did not progress, and at 3 weeks, azathioprine therapy (1.5 mg/kg) was initiated. After a 3-month oral prednisone taper and continuing azathioprine, the patient maintained 20/20 visual acuity in both eyes during their 10-month follow-up period [14].

Additionally, Providencia et al. described a unilateral serpiginous choroiditis 4 weeks after a PCR-confirmed COVID-19 infection. The patient received pulses of methylprednisolone 1 g/day for 3 days with subsequent tapering of the dose. Methotrexate 12.5 mg weekly was started simultaneously. During the follow-up, the patient’s vision improved from counting fingers to 20/100 with persistent central scotoma [15].

APMPPE is a rare, self-limited disorder and although the exact etiology of APMPPE remains unclear, it is thought to be an immune-mediated process with a possible infectious trigger. In order to establish the most likely cause in a given patient, detailed medical history and a methodical workup is crucial excluding all relevant infectious causes. Since the specific cause is difficult to ascertain in many cases, authors often define the disease as an idiopathic condition. We refrained from declaring our presented case idiopathic due to reasons explained earlier. Several case reports have described the association of APMPPE with various viral and bacterial infections [16–18]. APMPPE has also been described in connection with vaccinations i.e., varicella, hepatitis A, hepatitis B, meningococcal C, yellow fever, typhoid, and influenza [19–21] and recently there have been a few case descriptions following COVID-19 vaccination [22–24]. The disease has also been described in cases with pre-existing autoimmune and autoinflammatory conditions such as psoriasis, sarcoidosis, erythema nodosum, eczema, thyroiditis, granulomatosis with polyangiitis, polyarteritis nodosa, nephritis, ulcerative colitis, central nervous system vasculitis [1, 25, 26]. The risk of developing APMPPE may be influenced by genetics, as there have been reports of associations between certain genetic haplotypes such as HLA-B7 and HLA-DR2 [27].

APMPPE primarily affects the choriocapillaris and inner choroid, resulting in secondary changes to the retinal pigment epithelium (RPE) and outer retina [1]. The immune-mediated destruction of the RPE results in dysfunction of the blood-retinal barrier, allowing inflammatory cells and proteins to infiltrate the retina. This leads to the formation of the characteristic placoid lesions, which represent the accumulation of inflammatory cells and debris [1].

The hallmark clinical feature of APMPPE is the presence of multiple, yellow-white placoid lesions located in the posterior pole of the eye. These lesions are typically bilateral and symmetric, with a predilection for the macular area. Fluorescein angiography reveals early hypofluorescence due to blockage from the placoid lesions, followed by late staining due to leakage from the choroidal vessels. Optical coherence tomography (OCT) demonstrates hyperreflective lesions from the outer plexiform layer to the RPE often with disruption of the outer retinal layers and RPE and loss of the ellipsoid zone at the site of the placoid lesions [1]. These characteristics have been well demonstrated by the initial presentation of our patient in this report.

In contrast to APMPPE, the lesions observed in RPC are often more numerous, with a range of 50 to 100, and can be found throughout the posterior pole, mid-periphery, and far-periphery. These lesions may predate or occur simultaneously with macular involvement. RPC is characterized by the widespread distribution of both active and healed lesions scattered across the fundus, leading to a prolonged progressive and relapsing clinical course [2] The development of pigmented chorioretinal atrophy is a typical feature of RPC as the lesions heal [3].

Progression in APMPPE is not unheard of, in a retrospective analysis of a cohort of 86 patients with SC by Gupta et al., it was found that over the course of several months to years, 20 patients who initially presented with a clinical picture resembling APMPPE had progressed to SC [28].

The exact mechanisms by which SARS-CoV-2 affects the eye are still not fully understood. It has been suggested that COVID-19 may cause tissue damage by directly infecting cells through the angiotensin-converting enzyme 2 (ACE-2) receptor in the presence of transmembrane serine protease 2 (TMPRSS2) [29]. In the eye, ACE-2 receptors are expressed in the retinal ganglion cell layer, inner plexiform layer, inner nuclear layer, and photoreceptor outer segments, TMPRSS2 is expressed in multiple retinal neuronal cells, vascular and perivascular cells, and in retinal Müller glial cells [30]. Once the virus enters the eye, it can cause an inflammatory response, leading to various ocular manifestations [7, 29].

At present, there is no standardized treatment protocol for APMPPE, and mild, self-limiting cases may not require treatment. However, systemic corticosteroids have been the mainstay of treatment in most cases with neurological involvement, in cases where vision is considered threatened or when RPC is suspected [31, 32] They have been shown to result in resolution of visual symptoms and retinal lesions in the majority of patients. In addition, immunosuppressive agents, such as azathioprine, mycophenolate mofetil, cyclosporine or adalimumab, have been used in refractory cases. [12, 33, 34] Nonetheless, treatment should be tailored to each individual patient's disease course and response to therapy.

There is very limited evidence regarding the use of adalimumab in the treatment of APMPPE. However, a report of two cases of RPC by Asano et al. described adalimumab to be effective in managing the disease and allow the systemic corticosteroid to be tapered without any relapses [33] The rationale for initiating steroid-sparing therapy in our patient was the suspicion of RPC and the need for continued moderate-dose prednisolone treatment. Although methotrexate or mycophenolate mofetil are most commonly used as first-line agents in our clinic, adalimumab was chosen based on the rheumatological consultation and the discussion with the patient.

## Conclusions

We report a case of a bilateral APMPPE two months after a SARS-CoV-2 infection with subsequent progression to RPC and a long but full visual recovery and good response to adalimumab therapy. This case underscores the possibility of long-lasting activity of APMPPE following COVID-19. The scarcity of reports compared with the number of confirmed COVID-19 infections worldwide suggests a rare entity. The association of APMPPE with a variety of infections may suggest a common immunological aberrant response that might be triggered by various factors. Further examinations and case reports are needed to understand the role of biological therapy in the treatment of such cases.

### Supplementary Information

**Additional file 1: Table 1. **CARE checklist.

## Data Availability

Data sharing is not applicable to this article as no datasets were generated or analyzed during the current study.

## Abbreviations

APMPPE: Acute posterior multifocal placoid pigment epitheliopathy
RPC: Relentless placoid chorioretinopathy
SC: Serpiginous chorioretinitis
COVID-19: Coronavirus disease 2019
SARS CoV-2: Severe acute respiratory syndrome coronavirus 2
PCR: Polymerase chain reaction
BCVA: Best corrected visual acuity
OCT: Optical coherence tomography
FA: Fluorescein angiography
ICGA: Indocyanine-green angiography
ACE-2: Angiotensin-converting enzyme 2
TMPRSS2: Transmembrane serine protease 2
